# Loss-of-Function in SMAD4 Might Not Be Critical for Human Natural Killer Cell Responsiveness to TGF-β

**DOI:** 10.3389/fimmu.2019.00904

**Published:** 2019-05-01

**Authors:** Lachlan P. Healy, Gustavo R. Rossi, Jai Rautela, Charlotte A. Slade, Nicholas D. Huntington, Ingrid M. Winship, Fernando Souza-Fonseca-Guimaraes

**Affiliations:** ^1^Genetic Medicine and Family Cancer Clinic, Royal Melbourne Hospital, Parkville, VIC, Australia; ^2^Department of Medicine, The University of Melbourne, Parkville, VIC, Australia; ^3^Division of Immunology/Molecular Immunology, Department of Medical Biology, The Walter and Eliza Hall Institute of Medical Research, University of Melbourne, Parkville, VIC, Australia; ^4^Department of Cell Biology, Universidade Federal do Paraná, Curitiba, Brazil; ^5^Department of Clinical Immunology and Allergy, The Royal Melbourne Hospital, Melbourne, VIC, Australia; ^6^The Jeffrey Modell Diagnostic and Research Centre for Primary Immunodeficiencies, Melbourne, VIC, Australia; ^7^The Department of Biochemistry and Molecular Biology, Biomedicine Discovery Institute, Monash University, Clayton, VIC, Australia; ^8^Faculty of Medicine, Dentistry and Health Sciences, University of Melbourne, Melbourne, VIC, Australia

**Keywords:** SMAD4, loss-of-function mutations, Hereditary Hemorrhagic Telangiectasia, NK cell, TGF-β signaling, CD56^bright/dim^ subsets

## Abstract

We characterized the NK cell phenotype and function in three family members with Hereditary Hemorrhagic Telangiectasia (HHT) due to heterozygous *SMAD4* mutations. Loss-of-function mutation in this gene did not induce developmental effects to alter CD56^bright^ or CD56^dim^ NK cell subset proportions in peripheral blood; and did not result in major differences in either their IL-15-induced proliferation, or their cytokine secretion response to TGF-β1. These data suggest that *SMAD4* plays a redundant role in downstream TGF-β signaling in NK cells.

## Introduction

Natural killer (NK) cells are bone marrow-derived innate lymphocytes that are abundant in blood and lymphoid tissues and possess spontaneous anti-tumor activities (1). These activities are triggered by engagement of receptors to stress-induced ligands and lack of engagement of inhibitory self-ligands (2). The role of NK cell immunosurveillance in homeostasis is critical to identify early transformed cells, to prevent metastasis of circulating tumor cells to different body compartments (3, 4). The physiology of the inhibitory NK cell checkpoints is currently under investigation and is being targeted for translational immunotherapeutic development (5). Both murine and human NK cells are dependent on gamma chain cytokine signaling (e.g., IL-2 or IL-15) for their development, differentiation, homeostasis, and priming (6). In contrast, TGF-β signaling was shown to override NK cell metabolism in both species to inhibit their effector functions (7, 8). In addition to inhibiting effector functions, TGF-β signaling was also shown to upregulate tissue residency features in NK cells, suggesting its role in potentially differentiating conventional NK cells into more pro-angiogenic and less cytotoxic innate lymphoid cell (ILC)1-like cells (9, 10).

The TGF-β superfamily is a well-conserved pathway and is highly homologous across different species. The canonical signal transduction from the TGF-βRI/II heterocomplex phosphorylates SMAD2 and 3 and activates a signal transduction mediated by a complex assembled by several potential hetero/homo-combinations of phosphorylated SMAD2 and SMAD3. Subsequent interactions with the molecule SMAD4 translocate the complex to the cell nucleus to induce transcriptional activity (11). Although it is not a receptor / substrate, SMAD4 is believed to be necessary to aid pSMAD2/3 -triggered TGF-β gene responses. However, the specific function that SMAD4 adds to the transcriptional complexes is still unclear (12).

In humans, Hereditary Hemorrhagic Telangiectasia (HHT) results from autosomal dominant loss-of-function mutations for specific components of the TGF-β superfamily: *ENG*, encoding endoglin (HHT1), *ACVRL1* (HHT2), or more rarely *SMAD4*. Patients with HHT typically present with mucosal telangiectasia resulting in epistaxis, gastrointestinal bleeding, and anemia. Other clinical features include multi-organ arteriovenous malformations, and colonic polyps (commonly observed only in SMAD4-HHT patients). Under clinical management by specialized services, these patients can have a relatively normal lifespan (13). Studies phenotyping immune cell development in these patients are yet to be comprehensively performed. One recent report characterizing functions of IL-2-expanded NK cells from a patient with a *SMAD4*-loss-of-function mutation, demonstrated augmented response to TGF-β signaling and suggested an unexpected inhibitory role of SMAD4 in TGF-β signaling (14). However, observations from a single patient case report cannot be reliably generalized.

In this current report, we studied different aspects of NK cell phenotype and function from a mother, son and daughter with HHT classified as SMAD4-loss-of-function. Complementary to Cortez et al. we aimed to access a larger number of steady-state observations from different NK cell subsets, and to characterize responsiveness to TGF-β1 *in vitro* in a number of different assays, ranging from proliferation to cytotoxicity and cytokine production. We observed a number of parameters that suggest that SMAD4 plays a redundant role into responsiveness to TGF-β1 in human NK cells, with mutated cells displaying minimal differences in numbers, subset proportions, proliferation, cytotoxicity, and cytokine production along different maturation phenotypic stages.

## Case Reports

Patient HHT 1949F, a 69-years-old woman, had suffered from minor epistaxis and major bowel symptoms, mainly constipation, since her teenage years. At age 37 years she underwent partial colectomy for colonic cancer arising in a polyp. An episode of hematemesis from a bleeding gastric polyp necessitated partial gastrectomy, and she is now prone to recurrent hypoglycemic episodes. There was no history of frequent infective episodes, and she reported normal wound healing. Her father suffered from frequent and copious nosebleeds and died from a cerebrovascular event aged 56 years. A diagnosis of Juvenile Polyposis/Hereditary Hemorrhagic Telangiectasia (JP/HHT) was confirmed by identification of a frameshift mutation in *SMAD4* (NP_005350.1:p.Ser232GInfs^*^3), leading to a premature stop codon. Her son (Patient HHT 1965M,) and daughter (Patient HHT 1967F, described below have both inherited the SMAD4-mutation.

Patient HHT 1965M, aged 53, is the son of the above, inherited the same SMAD4 mutation. He underwent Whipple's surgery in his early 20s, for upper GI bleeding from extensive polyps. At 46- years of age, he suffered large bowel intussusception from polyps. Recent identification of significant iron deficiency anemia led to extensive endoscopic procedures including antegrade push enteroscopy, colonoscopy, and Pill-cam surveillance. Multiple ulcerated jejunal polyps were removed endoscopically, though many remain. Three polyps were also removed from the descending colon, the rectum, and the anorectal verge. Other significant past history included five episodes of pneumonia, starting in childhood.

Patient HHT 1967F, aged 51, daughter of HHT 1949F, experience significant skeletal pain and deterioration of bones and teeth. She suffered from numerous co-morbidities since childhood, including abdominal pain and rectal bleeding. She has recurrent kidney stones and previous pyleonephritis. Ongoing blood loss requires frequent iron infusions, and she undergoes SMAD4 mutation-related active surveillance for bowel cancer.

The patients above were coded in this study as HHT-1 (HHT-D 1956-M), HHT-2 (HHT-C 1967-F) and HHT-3 (HHT-D 1949F). Blood samples from three healthy donors were used controls: HD1 (male, 42 years old), HD2 (male, 28 years old), and HD3 (female, 54 years old).

## Materials and Methods

### Reagents

Commercial antibodies and reagents to detect human epitopes and stimulating cytokines used in this study are listed below:

- Abcam (Cambridge, MA): SMAD4 (EP618Y), beta Actin (mAbcam 8226).
- BD Biosciences (San Jose, CA): Annexin V-FITC / Apoptosis Detection Kit, Fixable Viability Stain (FVS) and Liquid Counting Beads.
- Biolegend (San Diego, CA): CD56-PE-Cy7 (HCD56), CD16-eFluor450 (3G8), CD62L-PE-CF-610 (DREG-56) and T-bet-PerCP (4B10).
- eBioscience (San Diego, CA): CD44-PE (IM7), and Eomes-eFluor660 (WD1928).
- Invitrogen (Carlsbad, CA): 123count Counting Beads, and Cell Trace Violet Cell Proliferation Kit.
- Miltenyi Biotec (Bergish Gladbach, Germany): CCR7-PerCP-Vio700 (REA546), CD8-VioBlue (REA734), CD45-VioGreen (5B1), CD49a-APC-Vio770 (TS2/7), CD49e-PE (NKI-SAM1), NKp46-APC (9E2), Propidium Iodide (PI) Solution, recombinant human IL-12 and human IL-15.
- MBL International (Woburn, MA): Recombinant human IL-18.
- Peprotec (Rocky Hill, NJ): Recombinant human TGF-β1.
- R&D Systems (Minneapolis, MN): Human IFN-γ, human GM-CSF and TGF-β1 Duoset ELISA Kits.
- Stem Cell Technologies (Vancouver, BC, Canada): EasySep Human NK cell Isolation Kit.

### Patients

Inclusion required a clinical diagnosis of HHT, and confirmation of the causative mutation.

### NK Cell Preparations and Culture Conditions

Heparinized peripheral blood (~30 mL) was obtained for each patient or healthy age-matched donor and processed by Ficoll-Paque density (1.077 g/mL) centrifugation, to isolate peripheral blood mononuclear cells (PBMCs) and plasma (for posterior TGF-β1 ELISA detection) from the red blood cell (RBC) fraction. NK cells from PBMCs were enriched by negative selection using the EasySep Human NK cell Isolation Kit (Stem Cell Technologies) for functional *in vitro* assays. PBMC fraction was also stained for either cell surface and intracellular markers, or only for cell surface markers for cell analysis using a BD FACS Fusion (BD Biosciences). Enriched NK cell subsets (final cell purity above 95%) isolated by negative selection for *in vitro* functional assays were maintained in RPMI 1640 media supplemented with 10% FCS, 5% human serum from male AB (Sigma-Aldrich, St. Louis, MO), 1% sodium pyruvate (Gibco, Grand Island, NY), 1% Glutamax (Gibco), 10 mM HEPES, 0.1% 2-mercaptoethanol (Gibco), 1% penicillin/streptomycin, and the indicated concentrations of cytokine stimulation accordingly for each assay.

### GM-CSF and IFN-γ Production, and T-Bet and Eomes Expression

High purity NK cells (CD3^neg^, CD4^neg^, CD8^neg^, CD14^neg^, CD20^neg^, CD66b^neg^, NKp46^+^, CD56^+^) were plated at 6 × 10^3^ cells per well in a V bottom microplate containing a final amount of 100 μL culture media per well. rIL-12 ([at]_F=_ 1 ng/mL), rIL-15 ([at]_F=_ 50 ng/mL) and rIL-18 ([at]_F=_ 50 ng/mL) were added to the cultures with or without rTGF-β1 ([at]_F=_ 6.25 ng/mL). After 48 h culture, the microplate was centrifuged for recovering supernatant for GM-CSF and IFN-γ ELISA. Cell pellets were washed 3x in cold PBS, then stained for intracellular T-bet and Eomes and analyzed using a BD FACS Verse (BD Biosciences).

### Target: Effector Cell Co-cultures

For cytotoxicity assays, fresh sorted NKp46^+^CD56^+^ NK cells (as described above) were incubated with 5 μM cell trace violet (CTV) (Thermo Fisher Scientific) according the manufacturer's instructions and cultured for 48 h in media containing rIL-15 ([at]_F=_ 50 ng/mL), with or without rTGF-β1 ([at]_F=_ 6.25 ng/mL), and then used to perform a 4 h co-culture assay using K562 target cells in a 1:4 ratio [ratio as described in Cortez et al. (14)] in a 96-well V bottom microplate. After 4 h co-culture, cell pellets were stained with Annexin V and PI (BD Biosciences) according the manufacturer, and cells were assessed on a BD FACS Verse cytometer (BD Biosciences), flow cytometric analysis was performed using Flowjo (Treestar, Ashland, OR) software. Target cell death was determined by apoptotic cells (Annexin V^+^), and necrotic cells (PI^+^) from the CTV^neg^ cell gate, and compared with a plate containing K562 cells without NK cells.

### Cell Proliferation

For proliferation assays, fresh sorted CD56^dim^ NK cells (as described above) were incubated with CTV according to the manufacturer's instructions, and 1 x 10^4^ labeled cells were seeded into 96-well V-bottom plates in culture media (200 μL/well) supplemented with rIL-15 ([at]_F=_ 50 ng/mL), with or without rTGF-β1 ([at]_F=_ 6.25 ng/mL). Time points (0, 40, 84, and 132 h) were assessed on a BD FACS Verse cytometer (BD Biosciences), flow cytometric analysis was performed using FlowJo (Treestar, Ashland, OR) software, and division numbers were determined using the precursor cohort-based method (15, 16).

### Statistical Analysis

Statistical analyses (as shown in the Figure legends) were performed using GraphPad Prism 7 software.

### Ethics

This study was carried out in accordance with approval of the Melbourne Health and Walter and Eliza Hall Institute of Medical Research's Human Research Ethics Committee (approval number: 2013.081). All subjects gave written informed consent for participation and publication.

## Results and Discussion

**Figure 1:**
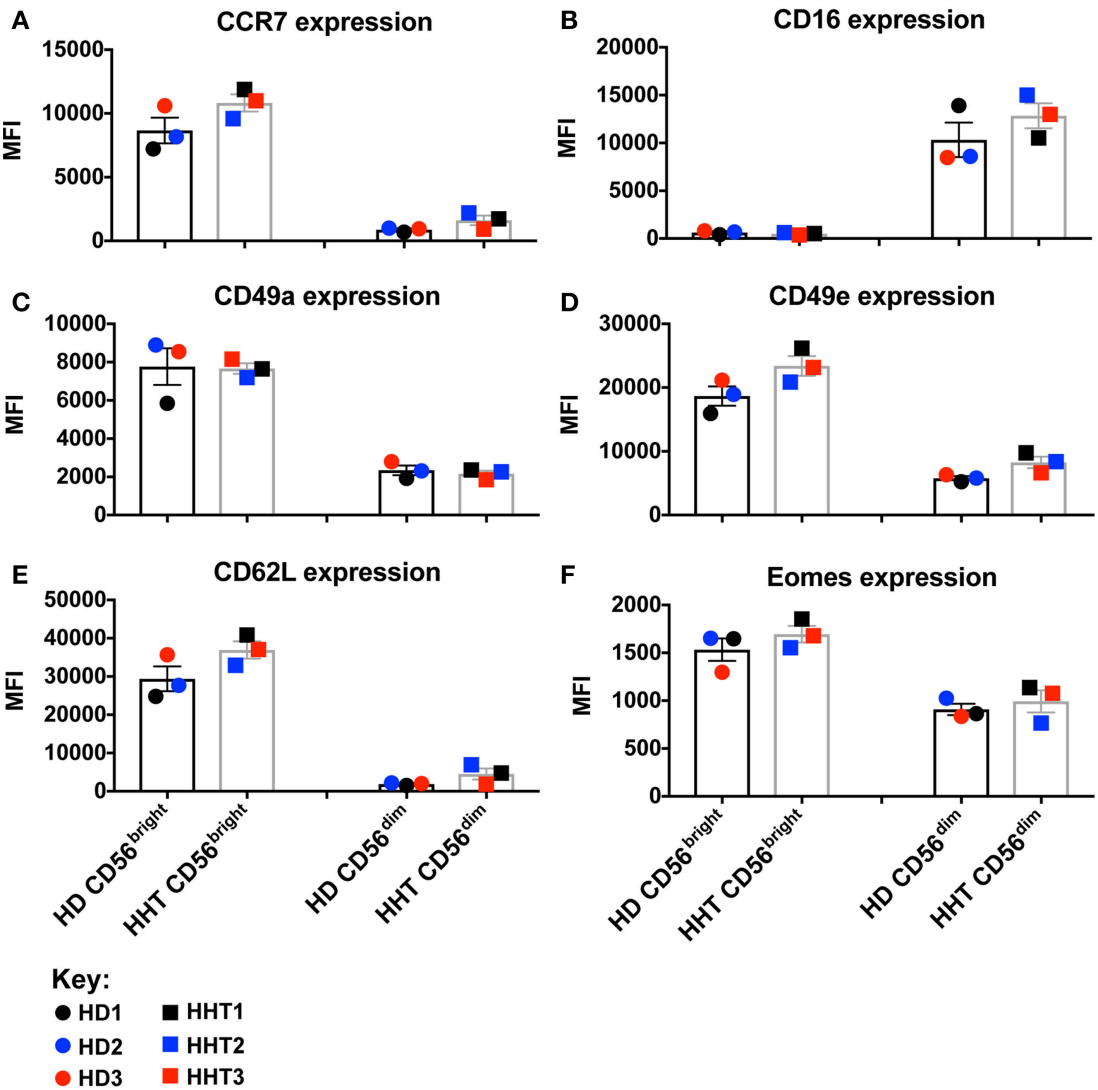
Surface and intracellular marker-characterization between CD56^bright^ and ^dim^ NK cell subsets from blood of HHT-SMAD4 donors. **A,B**: Peripheral NK cells (CD3^neg^, CD14^neg^, CD66b^neg^, CD20^neg^, NKp46^+^), from three SMAD4-mutated donors, and three healthy donors, were segregated according their level of CD56 expression (bright or dim) and analyzed for surface marker expression [CCR7 **(A)**, CD16 **(B)**, CD49a **(C)**, CD49e **(D)**, and CD62L **(F)**] and intracellular Eomes **(F)**. Circle dots represent health donors, and squares represent HHT donors. Color code was applied to represent the expression of each parameter in each donor alongside (black = donor #1, blue = donor #2 and red = donor #3). The respective numeric MFI (median fluorescence intensity) is represented on each overlay for each stain and donor. Unpaired *T*-test was used for comparative statistical analysis.

TGF-β signaling in NK cells is associated with: phosphorylation in SMAD2 and 3, inhibition of IL-15-induced metabolism/proliferation, simultaneous downregulation of CD44, CD49e, and Eomes, and upregulation of CD16 and CD49a expression (7, 10). SMAD family member 4 (SMAD4) belongs to the SMAD family of transcription factor proteins which combine in heterocomplexes with SMAD2 and 3, and still have an unclear role during the signal transduction (11, 12). In this case report, we obtained samples from patients carrying a loss-of-function mutation in the *SMAD4* (p.Ser232GInfs^*^3) expected to generate a protein without function and prone to be degraded upon expression. We accessed by western blot the SMAD4 protein expression, from fresh isolated NK cell by negative selection, two *SMAD4-*mutants and 2 health donors, and confirmed a lower expression of SMAD4 in both patient samples (data not shown). *SMAD4*-mutant and healthy donor NK cell phenotypes were assessed by gating the CD56^bright^ and CD56^dim^ subsets from the viable CD3^neg^, CD14^neg^, CD20^neg^, CD45^+^, CD66b^neg^, NKp46^+^ PBMC population. Although all donors were clinically classified as infection-free at time of donation, *SMAD4*-mutant CD56^bright^ and CD56^dim^ NK cells displayed decreased numbers in blood (Supplementary Figure 1). By overlaying the respective NK cell subsets (CD56^bright^ or ^dim^) from healthy donors (HD1, 2, and 3) and patients (HHT1, 2, and 3), multiparametric flow cytometry comparison of cell surface markers revealed no consistent/significant differences were observed in either of the SMAD4-deficient samples for CCR7, CD16, CD49a, CD49e, CD62L, and Eomes expression (Figures 1A–F, respectively, and Supplementary Figure 2). CD62L expression can subdivide CD56dim NK cells into a CD56^dim^CD62L^+^ population, which displays an intermediate maturation status between the CD56^bright^ NK cells (all CD62L^+^) and CD56^dim^CD62L^neg^ subsets (17). SMAD4-deficient donor HHT2 displayed a striking upregulation of CD62L in CD56^dim^ cells, but this effect was not seen in HHT1 or 3 who showed levels similar to the healthy donors (Supplementary Figure 2). Considering that TGF-β1 is a pleiotropic cytokine we also assessed if its levels were systemically increased at steady-state due to the clinical conditions associated with the *SMAD4* mutation. However, we could not observe any major increase of TGF-β1 in the plasma of SMAD4-deficient samples (data not shown).

**Figure 2:**
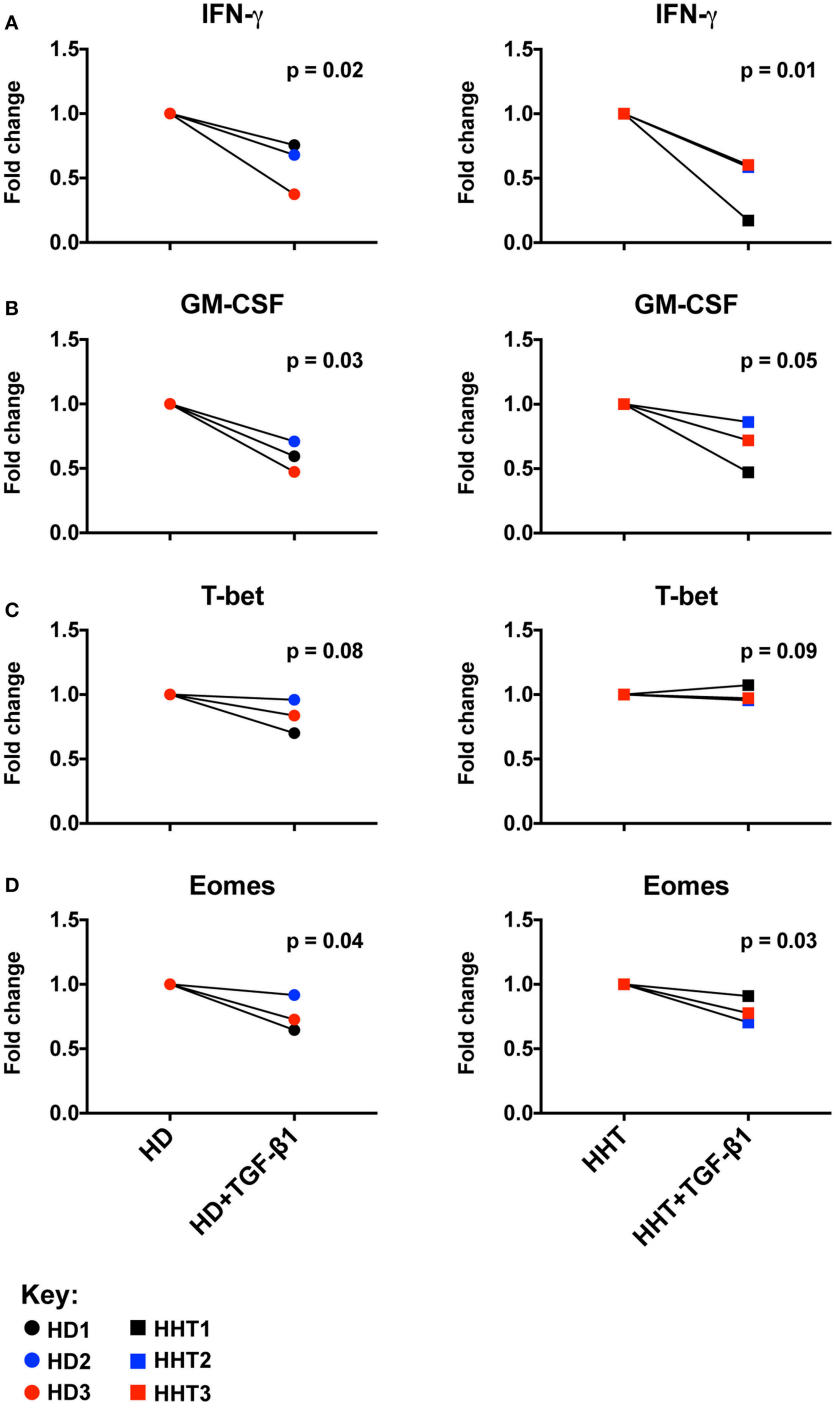
SMAD4-deficient NK cell subsets display normal Eomes, GM-CSF, IFN-γ expression patterns in response to TGF-β1. Purified NK cells, from three SMAD4-mutated donors and three healthy donors, were segregated according fold change of Eomes, GM-CSF, IFN-γ, and T-bet expression after a 48 h *in vitro* stimulation under IL-12+IL-15+IL-18 with or without TGF-β stimulation to evaluate their respective potential to express IFN-γ **(A)** and GM-CSF **(B)** from culture supernatants, and intracellular T-bet **(C)** and Eomes **(D)** from cell pellets at endpoint. Results are expressed in fold change according unstimulated/TGF-β stimulated for each biological replicate of HHT and HD donors. Circle dots represent health donors, and squares represent HHT donors. Color code was applied to represent the expression of each parameter in each donor alongside (black = donor #1, blue = donor #2 and red = donor #3). Unpaired *T*-test was used for comparative statistical analysis.

To ascertain the role of SMAD4 in the NK cell, CD56 subsets were sterilely sorted by negative selection to achieve high purity, and compared in *in vitro* functional assays in the presence of activating cytokines and presence or absence of TGF-β1 to examine their responsiveness to this cytokine. Combined with IL-15, IL-12, and IL-18 efficiently upregulate and sustain expression of cytokines such as GM-CSF and IFN-γ in human NK cells (18, 19), in mechanisms governed by Eomes (to drive the NK cell maturation program) and T-bet (to drive transcriptional regulation of cytokine genes such as IFN-γ) (20). IFN-γ and GM-CSF expression was decreased in SMAD4-deficient NK cells from all subjects by TGF-β1-stimulation, similarly to health donors NK cells (Figures 2A,B; Supplementary Figure 3). By contrast, T-bet expression was not lowered, while Eomes was further inhibited in the presence of TGF-β1 (Figures 2C,D; Supplementary Figure 3). Considering that those effector molecules, among others, are involved in NK cell immunosurveillance and control of target tumor cells, we next speculated that the cytotoxicity function of the SMAD4-deficient NK cells might be differentially affected.

**Figure 3:**
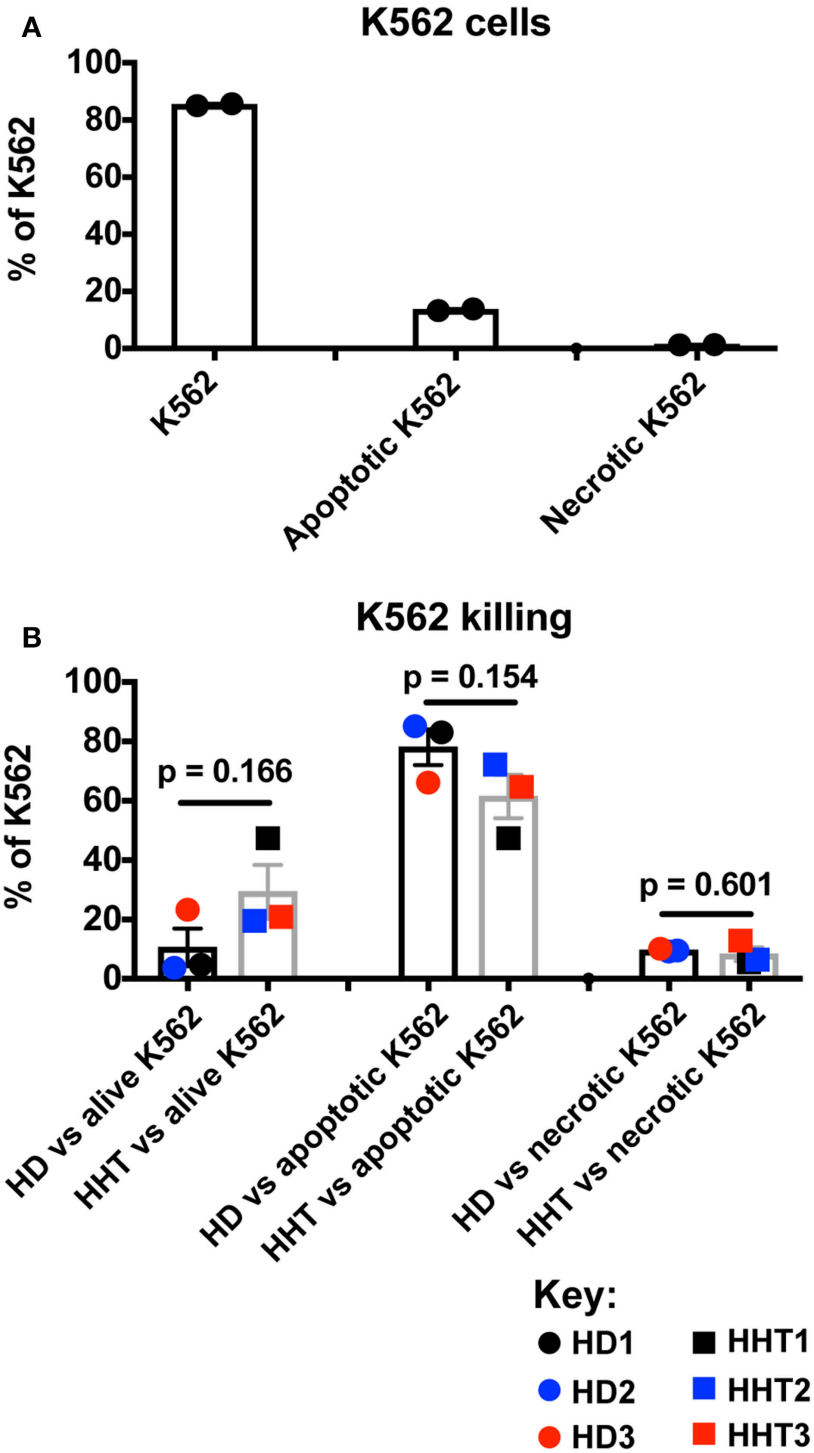
SMAD4-deficient NK cells display similar killing patterns of target K562 cells. *In vitro*-expanded K562 were stained for Annexin V and PI prior **(A)**, and after incubation for 4 h with purified NK cells previously treated for 48h with IL-15 **(B)**. Results are expressed as the ratios of alive K562 cells (Annexin V^neg^, PI^neg^), apoptotic K562 cells (Annexin V^+^), and necrotic K562 cells (PI^+^) for each respectively, culture condition as the mean + SEM of each biological replicates for HHT and HD samples. Circle dots represent health donors, and squares represent HHT donors. Color code was applied to represent the expression of each parameter in each donor alongside (black = donor #1, blue = donor #2 and red = donor #3). Unpaired *T*-test was used for comparative statistical analysis of the mean of the technical replicates of each donor.

Cortez et al. assessed IFN-γ production as an activation outcome of SMAD4-deficient NK cells from one donor after co-culture with K562 targets, therefore the target cell killing was not measured (14). To address the role of SMAD4 in cytotoxicity, we next sorted highly purified CD56^dim^ NK cells, the more cytotoxic subset [(21) and Supplementary Figure 3], from healthy and SMAD4-deficient donors and primed them with IL-15 prior to adding K562 targets in a 4:1 ratio as in the study of Cortez et al. By using the Annexin V and PI method of K562 apoptosis quantification (22), we first observed a high viability in target cell cultures without effector cells (Figure 3A), which was dramatically decreased after the addition of IL-15-primed control or SMAD4-deficient NK cells for 4 h (Figure 3B). However, live and death in K562 cells were not significantly changed in co-culture with *SMAD4*-deficient or WT NK cells in presence of IL-15-treated NK cells This result suggested that cytotoxicity of SMAD4-deficient NK cells is not suggested to be affected by the mutation.

**Figure 4:**
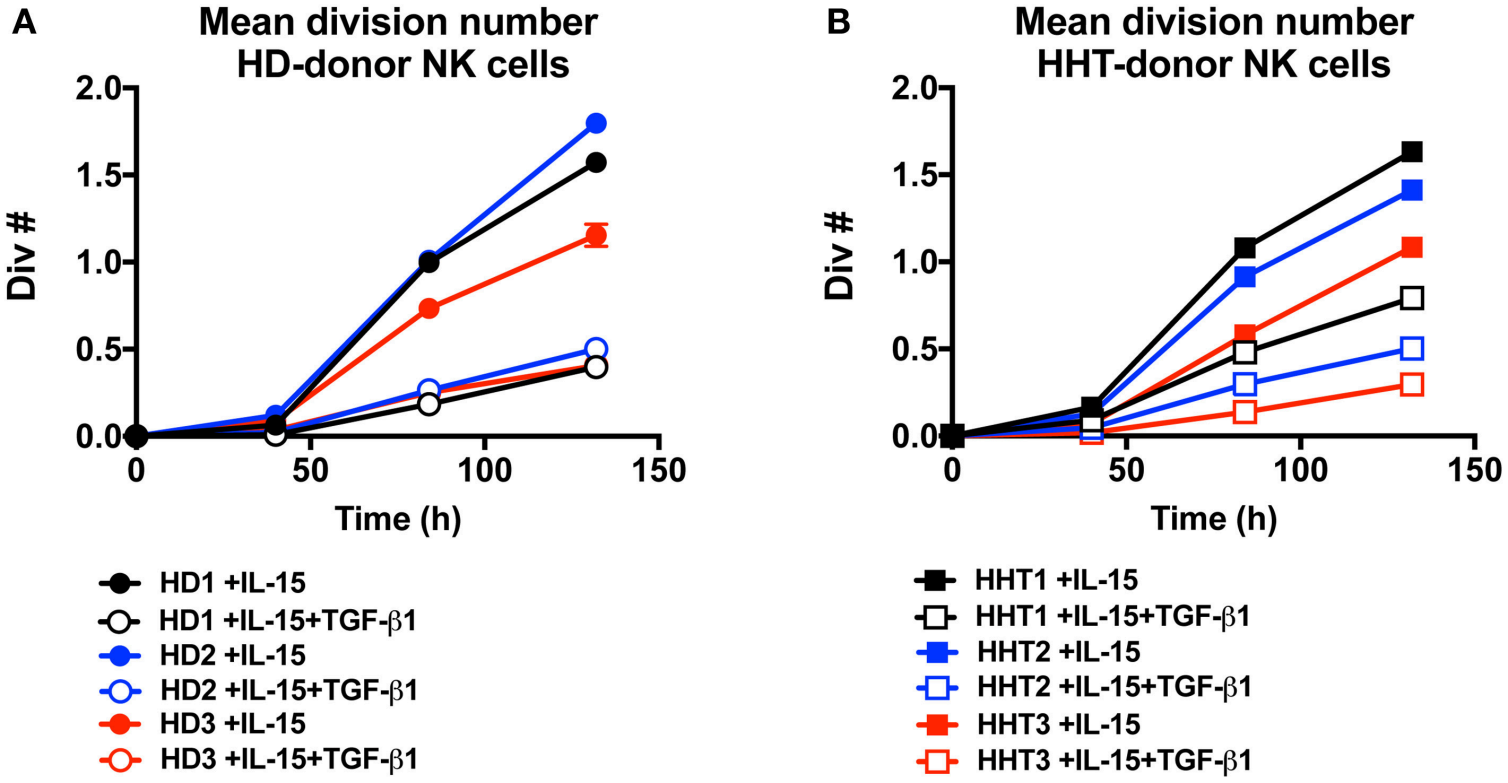
SMAD4-deficient NK cells display similar susceptibility to TGF-β-mediated inhibition of cellular proliferation as WT cells. CTV-labeled NK from three health donors **(A)** and three SMAD4-mutated donors **(B)**, were cultured in presence of rIL-15 and with or without addition of rTGF-β1 as indicated. For each condition at 40, 84, and 132 h intervals, the total live cells were enumerated and mean division number was calculated. Circle dots represent health donors, and squares represent HHT donors. Color code was applied to represent the expression of each parameter in each donor alongside (black = donor #1, blue = donor #2 and red = donor #3). Results are representative from triplicate of each biological replicate.

IL-15 signaling is a critical survival factor which also induces NK cell priming and proliferation. This is attracting great interest in manipulating this pathway to enhance NK cell function for immunotherapy development (5, 6). TGF-β signaling was previously shown by us and others to be an antagonistic pathway that efficiently represses IL-15-induced proliferation in NK cells (7, 23). We next examined whether SMAD4-deficient NK cells would display differential proliferation potential in response to IL-15 (Figures 4A,B). Conversely, TGF-β1 efficiently reduced the mean division number of NK cells from both healthy donors (Figure 4A), and SMAD4-defficient cells (Figure 4B). These results suggest that SMAD4 deficiency does not cooperate with SMAD2 and 3 to suppress IL-15-induced proliferation in human NK cells.

## Concluding Remarks

A limitation inherent in reports based on a single or few donors, is the risk of over-interpretation from a limited sampling size. SMAD4 mutations are rare, and obtaining a large sample cohort is challenging. In the current study we described the NK cell phenotype of three family members carrying the same loss-of-function *SMAD4* mutation. Although a previous study suggested that SMAD4 acts as an inhibitory molecule to the TGF-β signaling in human NK cells, our results suggests that this molecule plays a redundant role for human NK cell development and function. The clinical impact of *SMAD4* mutations on immunity has not been well-characterized, due to the rarity of the condition, and the predominance of other clinical features. Future studies using a larger cohort may determine the redundancy of specific signaling members of the TGF-β superfamily for NK cell biology, and help us understand the complexity of their roles in the immune status of patients carrying mutations in these genes.

## Ethics Statement

This study was carried out in accordance with approval of the Melbourne Health and Walter and Eliza Hall Institute of Medical Research's Human Research Ethics Committee (approval number: 2013.081). All subjects gave written informed consent in accordance with the Declaration of Helsinki, and donors provided informed consent for publication.

## Author Contributions

LH, GR and JR assisted with the performance of experiments. FS-F-G conducted the experiments and analyzed the data. NH, CS, IW, and FS-F-G interpreted the data and reviewed and revised the manuscript. LH, and IW genotyped, diagnosed, and recruited the patients for the study. LH, NH, IW, and FS-F-G co-wrote the manuscript, reviewed the manuscript, and agreed with all aspects of work. FS-F-G and IW designed and supervised the study. All authors approved the manuscript and agreed with all aspects of the work.

### Conflict of Interest Statement

NH and JR are co-founders and shareholders in oNKo-Innate. NH has a research contract with Servier. NH, JR, and FS-F-G have a funded research collaborative agreement with Paranta Bioscience Ltd. The remaining authors declare that the research was conducted in the absence of any commercial or financial relationships that could be construed as a potential conflict of interest.

## Supplementary Material

Supplementary Figure 1 CD56bright and CD56dim NK cell numbers in peripheral blood of health donors and HHT-SMAD4 subjects.

Supplementary Figure 2 CCR7, CD16, C49a, CD49e, CD62L and Eomes expression patterns between different health donors and HHT-SMAD4 subjects.

Supplementary Figure 3 IFN-gamma and GM-CSF secretion, a T-bet and Eomes expression comparison in NK cells from health donors and HHT-SMAD4 subjects.
