# Supplementary figures and images for: Loss-of-Function in SMAD4 Might Not Be Critical for Human Natural Killer Cell Responsiveness to TGF-β
Source: Front Immunol. 2019 May 1;10:904. doi: 10.3389/fimmu.2019.00904 (PMC6506781; doi:10.3389/fimmu.2019.00904)

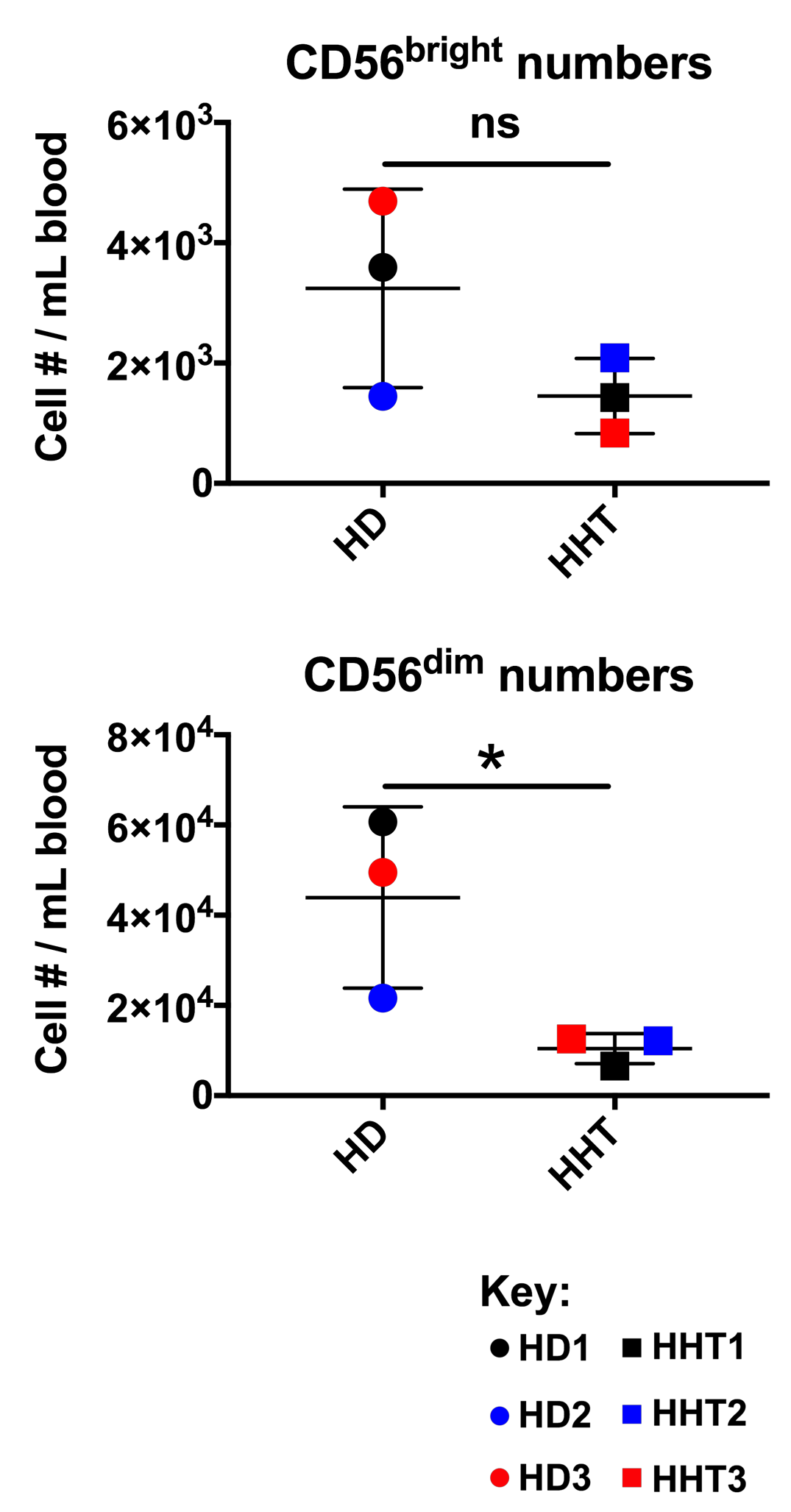

Supplement: Supplementary Figure 1 — CD56bright and CD56dim NK cell numbers in peripheral blood of health donors and HHT-SMAD4 subjects. [file Image_1.TIFF]

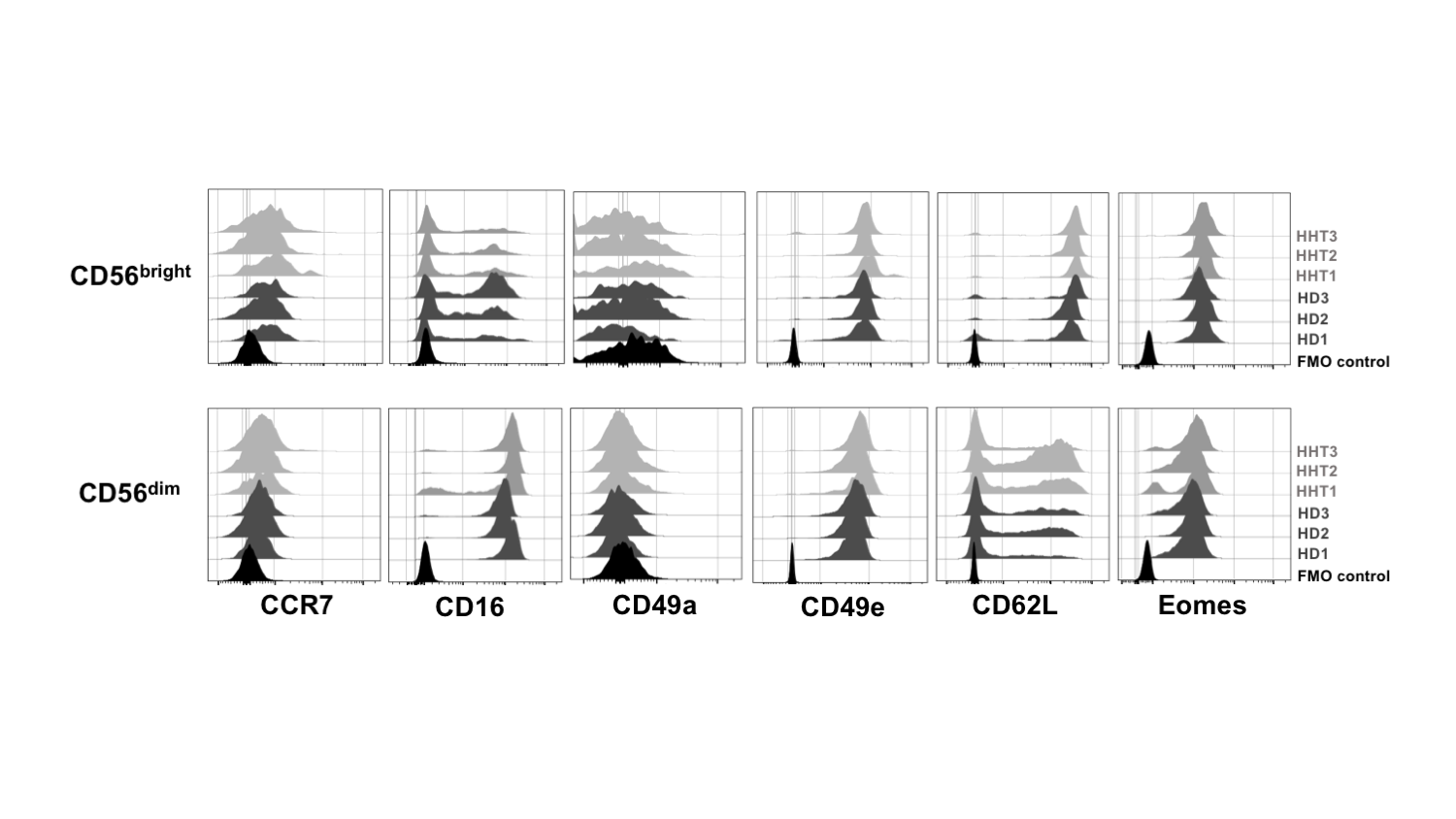

Supplement: Supplementary Figure 2 — CCR7, CD16, C49a, CD49e, CD62L and Eomes expression patterns between different health donors and HHT-SMAD4 subjects. [file Image_2.TIFF]

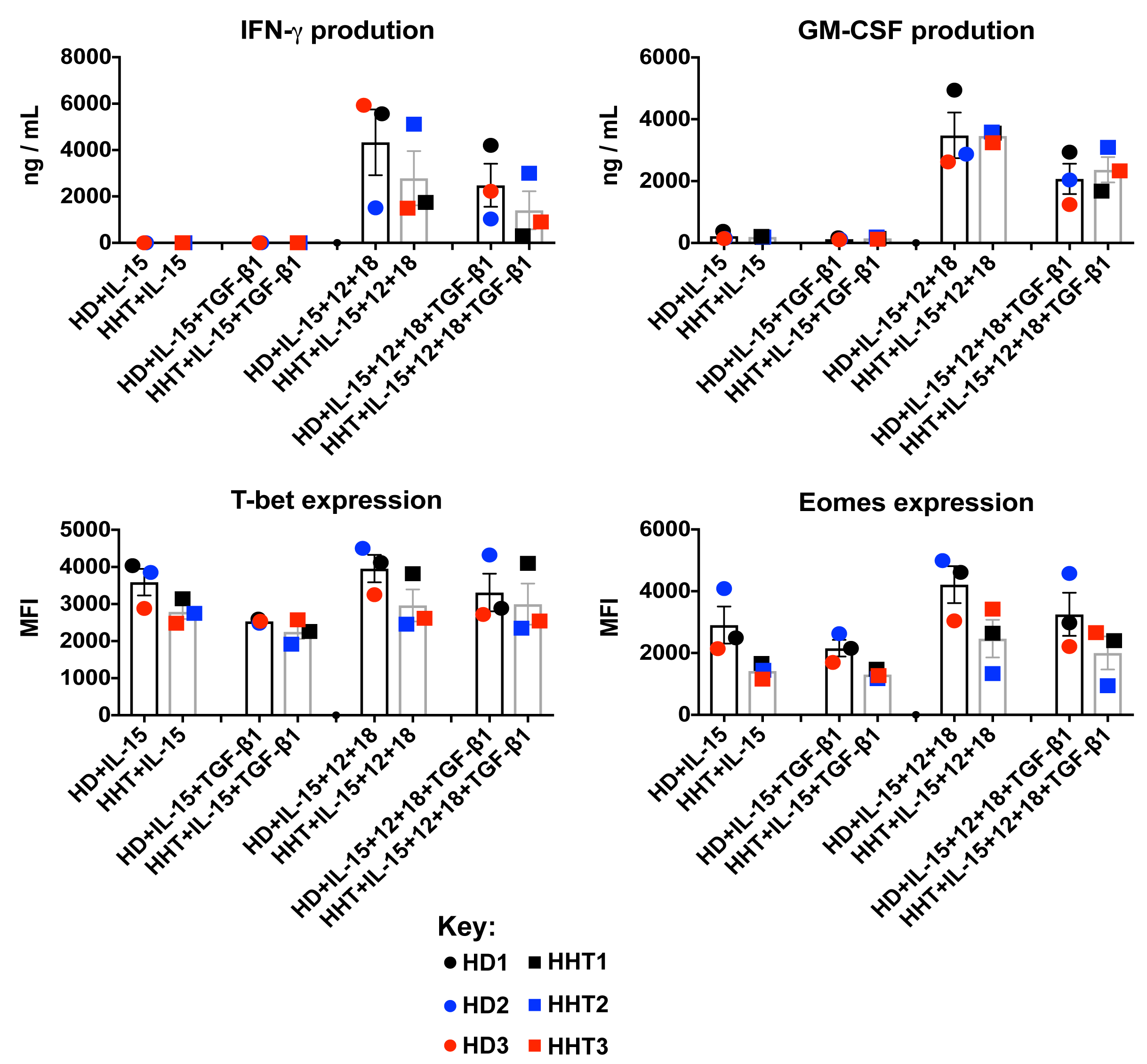

Supplement: Supplementary Figure 3 — IFN-gamma and GM-CSF secretion, a T-bet and Eomes expression comparison in NK cells from health donors and HHT-SMAD4 subjects. [file Image_3.TIF]
